# A case of atrial septal defect associated with anomalous sinoatrial node artery in pulmonary atresia with intact ventricular septum

**DOI:** 10.1111/echo.15054

**Published:** 2021-05-24

**Authors:** Angela Di Candia, Biagio Castaldi, Domenico Sirico, Giovanni Di Salvo

**Affiliations:** ^1^ Pediatric Cardiology Unit Department of Woman and Child’s Health University of Padua Padua Italy

**Keywords:** atrial septal defect, congenital heart disease, gore septal device, interventional cardiology, sinus node coronary artery

## Abstract

An 11‐year‐old boy affected by pulmonary atresia with intact ventricular septum (AP‐IVS) was listed for percutaneous pulmonary valvuloplasty and closure of multi‐fenestrated atrial septal defect (ASD). Intra‐procedural transesophageal echocardiography arose the suspect of abnormal coronary artery pattern while selective angiography documented a single sinoatrial node artery (SANa) with an unusual retro‐aortic course. As consequence, we proceeded to effectively close the defects with a not self‐centering device placed in the most central side hole. This case supports the hypothesis that sometimes arrhythmic complication during ASD closure procedures might be due to unrecognized injury of the SANa.

## 1 INTRODUCTION

The sinoatrial node artery (SANa) has significant anatomic variability and some variants have been described to be associated with a greater risk of iatrogenic injury during surgical or percutaneous procedures.

**FIGURE 1:**
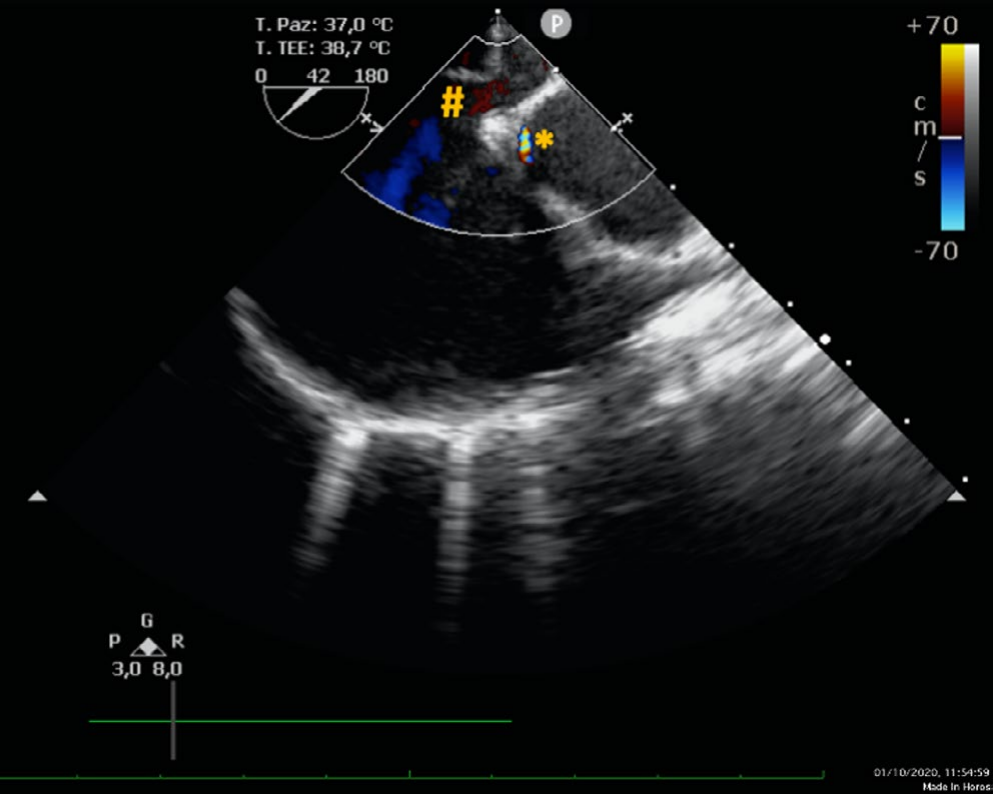
Aortic short axis transesophageal echocardiography showing main atrial septal defect (hash) and an unusual flow behind the noncoronary aortic sinus (asterisk)

**FIGURE 2:**
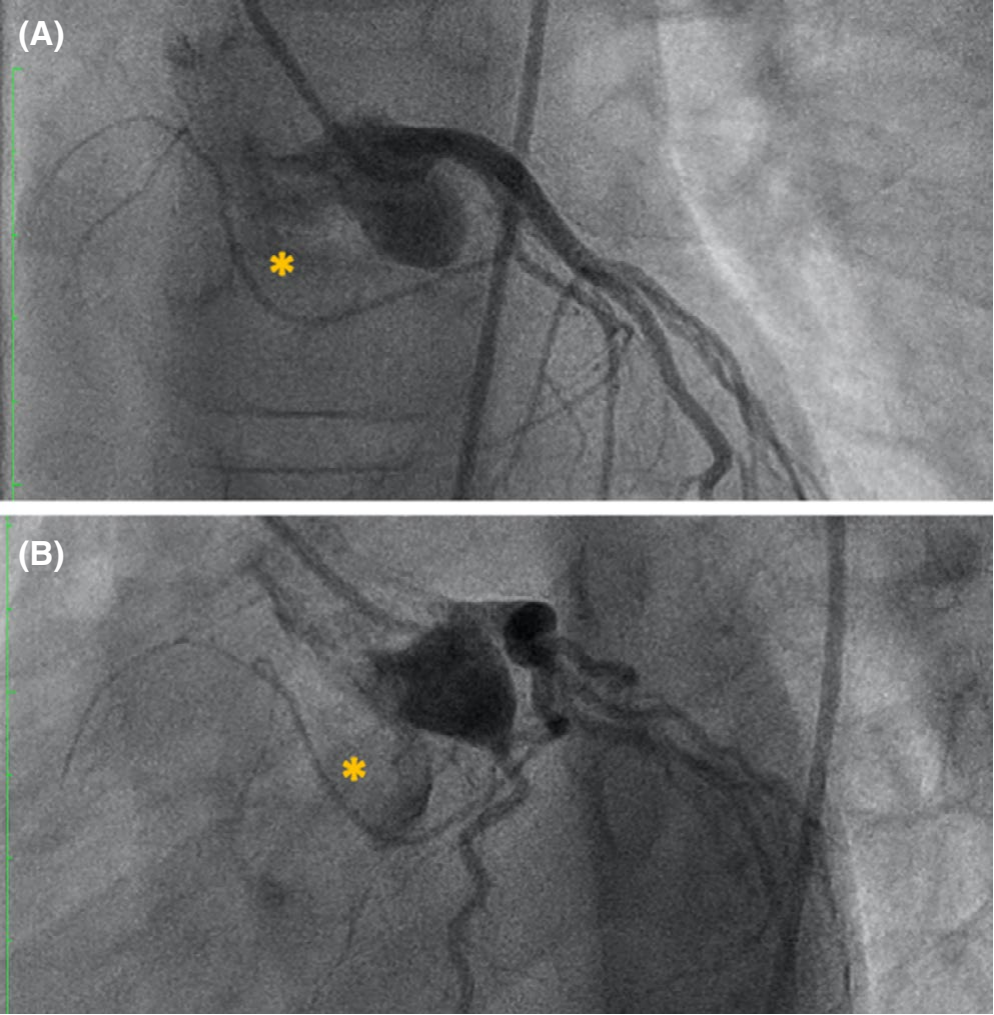
Antero‐posterior (a) and lateral (b) views of selective left coronary angiography. Sinoatrial node artery is marked by asterisk

An 11‐year‐old boy affected by pulmonary atresia with intact ventricular septum was evaluated at our center for a follow‐up visit. The patient underwent percutaneous pulmonary valve perforation and pulmonary valve dilatation in neonatal age. Right ventricular angiography excluded coronary sinusoids. Aortic root injection confirmed normal coronary artery anatomy and absence of coronary fistulas. The percutaneous pulmonary valve dilatation was repeated at 1 and 7 years of life. An atrial septal defect (ASD) was monitored over the time. Given the echocardiographic finding of significant gradient through the pulmonary valve and spontaneous right‐to‐left atrial shunt with evidence desaturation during exercise stress test, the boy was listed for the fourth percutaneous pulmonary valvuloplasty and percutaneous closure of multi‐fenestrated ASD. The largest ASD was located antero‐superiorly just behind the Aorta. A static sizing by using an 18 mm Amplatzer® Sizing Balloon (Abbott) showed a stretched diameter of 11 mm. In addition, a patent foramen ovale (PFO) and a small, more central, ASD were found. Intra‐procedural transesophageal echocardiography (TEE) showed an unusual flow behind the noncoronary aortic sinus, rising the suspect of abnormal coronary artery pattern (Figure 1, Video 1). For this reason, selective coronary angiography was performed. Left coronary angiogram documented a single SANa having a course compatible with the echocardiographic finding. Indeed, this artery appeared to originate from the left circumflex coronary artery (LCCA), turn posteriorly, run close to the noncoronary aortic cusp, cross the antero‐superior inter‐atrial groove, and continue with a precaval course toward the sinoatrial node (Figure 2). Coronary angiography highlighted the proximity relationship between the ASD and the SANa, the latter intersecting the antero‐superior rim of the inter‐atrial septum. On the basis of the hemodynamic data collected, after successful pulmonary valvuloplasty, we proceeded with the ASD closure by using a 30 mm Gore® Septal Occluder device (WL Gore & Associates) deployed in the central side hole rather than a self‐centering device in the larger antero‐superior ASD.

**VIDEO 1:** Aortic short axis transesophageal echocardiographic view, 42° ‐ mid esophageal plane. An unusual tiny systolic flow can be seen between the atrial septal defect and the aortic non‐coronary sinus. The full‐text HTML version of this article includes video content. To view this version please visit https://onlinelibrary.wiley.com/doi/10.1111/echo.15054

**FIGURE 3:**
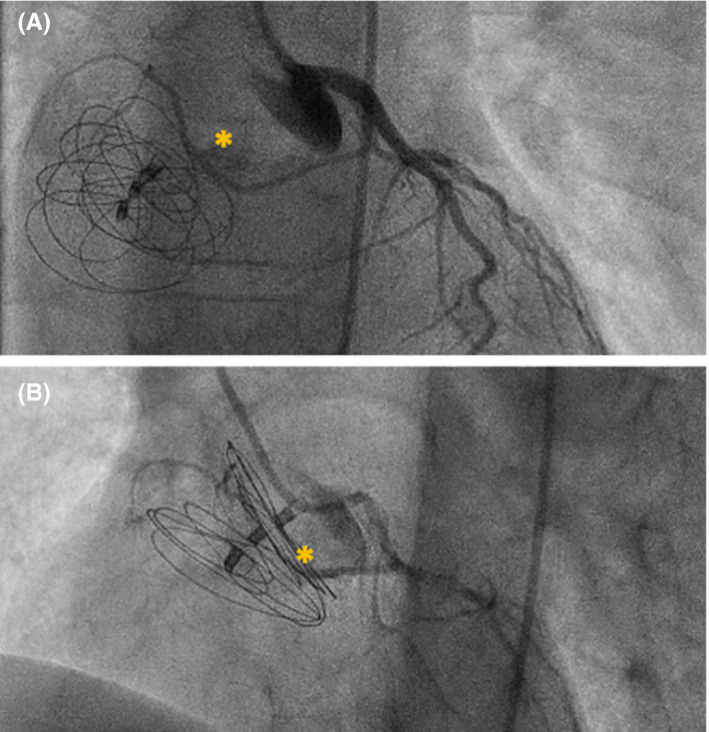
Antero‐posterior (a) and lateral (b) views of selective left coronary angiography after Gore® Septal Occluder device deployment. Sinoatrial node artery is marked by asterisk

At the end of the procedure, good conformation of the device and absence of residual shunts were documented. The left coronary artery (LCA) was rechecked by selective coronary angiography: the SANa ran adjacent to the device, without being touched by it (Figure 3). The ECG did not show any changes in the heart rhythm. The follow‐up was uneventful. After ASD closure, ASA 100 mg for 6 months was prescribed.

Pulmonary atresia with intact ventricular septum (PA‐IVS) is a rare congenital heart disease (3% of congenital heart disease, with an incidence of 4–8 out of 100 000 live births). It can be associated with anomalies in the development of the coronary arteries, linked to the peculiar pathophysiology that leads to the creation of ventriculo‐coronary fistulas or atretic coronary tracts.^1^ To our knowledge, no anomalies in the course of the SANa associated with PA‐IVS have been reported to date.^2^

Autoptic or imaging study described a wide anatomical variability of the SANa in the general population, regarding either the origin and the course.^3^, ^4^, ^5^, ^6^ Most commonly, SANa arises from right coronary artery (RCA; 54%‐68% of cases) or from LCCA (22%‐40% of cases). Regarding the relationship with the superior vena cava, SANa can present a retrocaval or a precaval or a pericaval course. The former is described to be about 47% of total cases and is the most frequent in the case of origin from the LCA/LCCA in the European population. The precaval and pericaval courses follow, representing respectively 39% and 14% of the total cases. High risk anatomical variants, such as S‐shaped artery, are described to be more associated with possible sinus node dysfunction during surgical or percutaneous procedures, which involve a manipulation of atrial walls or inter‐atrial septum.^3^, ^4^ They must be taken into account to prevent iatrogenic injuries. In fact, the damage of the SANa is associated with dysfunction of the sinus node and the onset of arrhythmias, such as junctional rhythm or supraventricular tachycardia.^7^, ^8^, ^9^

Saremi et al (2008) in a study of 250 patients undergoing coronary CT, described 13.9% of cases having the S‐shaped variant, which originated from LCCA, turned posteriorly and passed in a groove at the junction between the orifice of the left upper pulmonary vein and the mouth of the left atrial appendage. Only one third of these cases presented a precaval course.^4^

**FIGURE 4:**
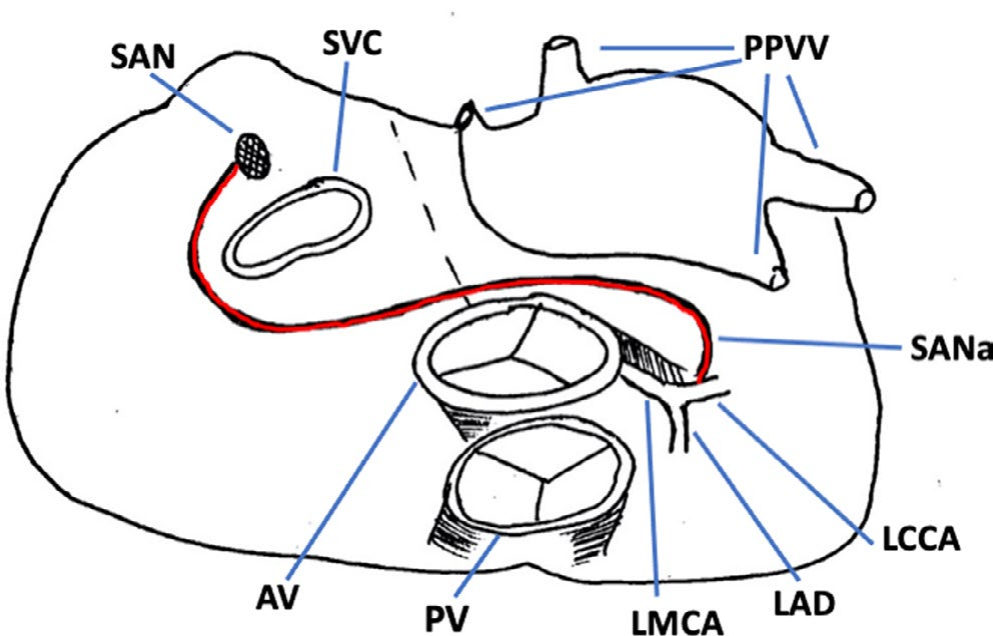
Example drawing of the sinoatrial node artery (in red) described in the case report. AV =aortic valve; LAD =left anterior descending coronary artery; LCCA =left circumflex coronary artery; LMCA =left main coronary artery; PPVV =pulmonary veins; PV =pulmonary valve; SAN =sinoatrial node; SANa =sinoatrial node artery; SVC =superior cava vein

In our case, conversely, the SANa didn't embrace the left atrial appendage and the superior left pulmonary vein: after detaching from the LCCA, our SANa immediately turned posteriorly with an acute angle towards the noncoronary aortic cusp, crossed the antero‐superior rim of the inter‐atrial septum and continued with a precaval course towards the atrial sinus node (Figure 4).

In conclusion, our case describes a new variant of SANa course, which crossed the antero‐superior groove of the atrial septum, found in a patient with AP‐IVS. Despite a coronary angiography is not routinely performed before ASD closure, a careful echocardiographic assessment of the defect, and the surrounding structure as well, is mandatory to minimize the risk of procedural complications.

Lastly, in case of ASD closure procedures complicated by rhythm alterations (0.6%‐2.7% of cases),^10^ such as junctional rhythm or supraventricular tachycardias, an unrecognized SANa course anomaly might be taken into account.

## CONFLICT OF INTERESTS

None.

## Data Availability

Data of the patient reported in this case are available after motivated request by e‐mail to the Corresponding Author.
